# Multiple Magnet Ingestion leading to Bowel Perforation: A Relatively Sinister Foreign Body

**DOI:** 10.7759/cureus.5866

**Published:** 2019-10-08

**Authors:** Muhammad Arshad, Sarah Masroor Jeelani, Areej Salim, Bakhtawar Dilawar Hussain

**Affiliations:** 1 Pediatric Surgery, Aga Khan University Hospital, Karachi, PAK

**Keywords:** pediatrics, emergency, foreign body, magnets, perforation

## Abstract

Foreign body ingestion is a common reason for seeking emergency care among children. One of the more serious foreign bodies are the ingestion of multiple magnets or concurrent ingestion of a magnet and a metallic foreign body. Conservative management with serial imaging can be misleading in such cases. Multiple magnets tend to have strong attractive forces among them and may encase loops of bowel within them. Once entrapped, pressure necrosis and perforation will ensue, and thus, a low threshold should be adopted for surgical exploration in such cases. We present the case of a two-year-old male who had an accidental, unwitnessed ingestion of multiple magnets and also report the subsequent surgical management and associated morbidity

## Introduction

Ingestion of multiple magnets is a serious health hazard for children, with an extremely high risk of intestinal obstruction and perforation in comparison with single magnet ingestion or other non-magnetic foreign bodies, the latter being most common. Magnets in plurality tend to capture loops of bowel in between them, which leads to localized necrosis and perforation. Sometimes multiple magnets may take certain forms and masquerade as other foreign bodies delaying surgical intervention [1]. The earliest report of bowel perforation with the ingestion of a traditional magnet was from Japan in 1995 [2]. Herein, we present a two-year-old male with un-witnessed ingestion of eleven magnets leading to gut perforation at multiple levels.

## Case presentation

A two-year four-month-old male presented to the emergency department with complaints of non-bilious, non-projectile vomiting for five days. He was initially treated with an antiemetic at a secondary care hospital. The vomiting continued until he was unable to tolerate oral feeding. He was then brought to the emergency department at our tertiary care. The child had no abdominal pain or fever upon presentation. He was constipated and had not passed stool in the last 24 hours but was passing urine. He had no past medical or surgical history. His immunization was up to date. On examination, the child was hemodynamically stable, while his abdomen was soft and non-tender. No visceromegaly was noted; however, there was fullness in the epigastric region with inaudible gut sounds.

**Figure 1:**
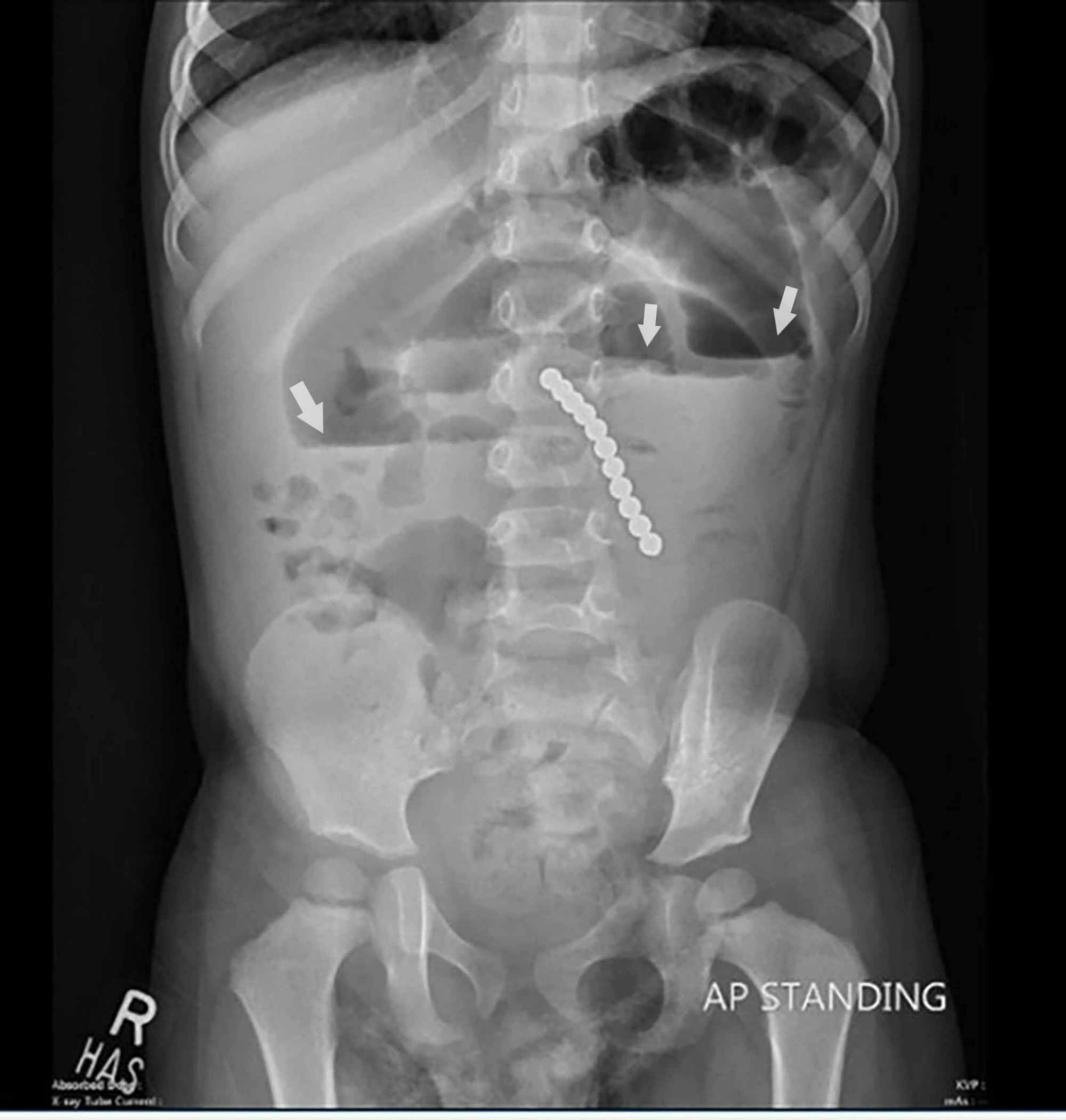
Well-defined chain of beaded radioopaque densities representing foreign body, projecting in the proximal small bowel loops with signs of small bowel obstruction Arrows indicate air-fluid levels.

Upon admission, his laboratory investigations revealed normal serum electrolyte levels, with a hemoglobin level of 12.0 mg/dl and a normal white blood cell count of 7.0 x 10^3^/microliters, with neutrophilia of 74.4%. An erect abdominal X-ray was performed, which revealed a well-defined chain-of-beads like radiopaque density projecting in the proximal small bowel loops, with air-fluid levels noted in the rest of the small bowel along with fecal loading appreciated within the large bowel. No signs of pneumoperitoneum were visualized (Figure 1).

A diagnosis of foreign body ingestion leading to bowel obstruction was made. A nasogastric tube was passed. On reviewing with the parents, they recalled the recent purchase of a magnetic toy for the child with similar-looking metallic beads. An emergent exploratory laparotomy was performed on the first day of admission. 

**Figure 2:**
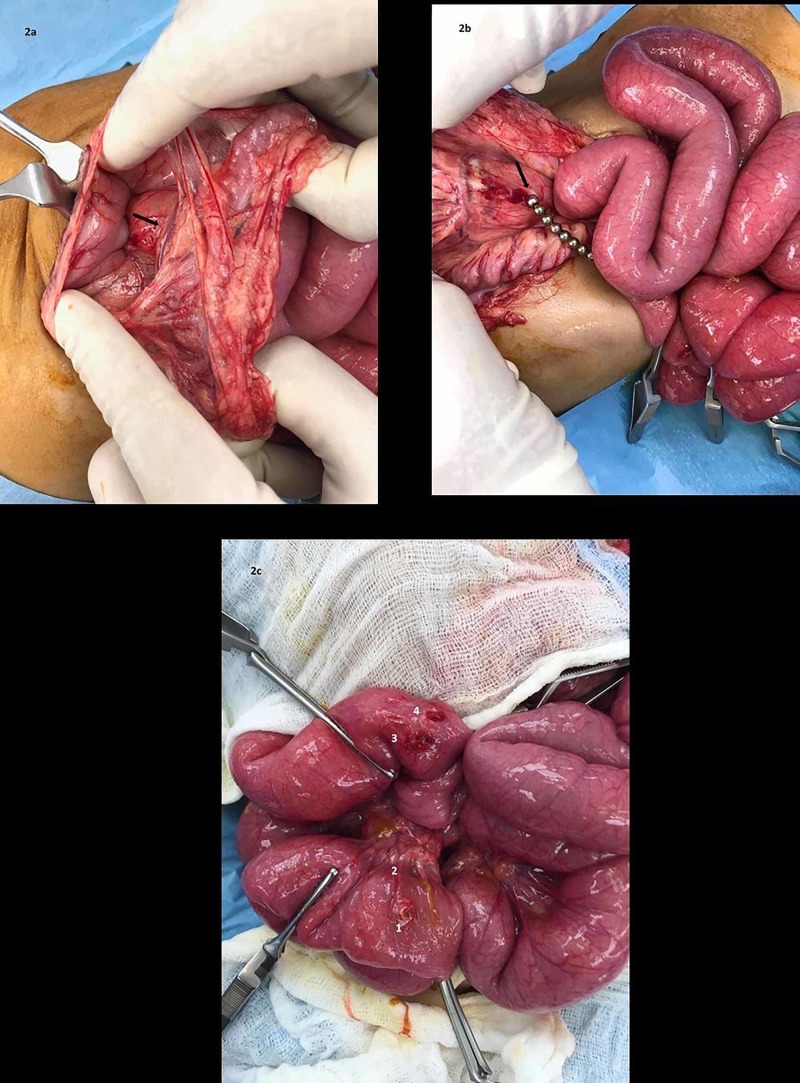
(a) Posterior wall of stomach adherent to transverse colonic mesentery secondary to magnetic attraction; (b) eight metallic beads noted, causing a transverse colonic mesenteric perforation due to attraction with three beads present in stomach; (c) small bowel perforations noted at four sites (1-4) secondary to the eleven ingested magnets

Intraoperatively, straw-colored fluid was observed within the abdominal cavity. The posterior wall of the stomach was adherent to the transverse colonic mesentery secondary to the magnetic force of the beads among bowel loops (Figure 2). A perforation of around 0.5 cm was noted in the posterior wall of the stomach; eight metallic beads were found in between small bowel loops and three beads within the stomach (Figure 2). Four full-thickness perforations were noted in the small bowel, 110 cm distal to the duodenojejunal (DJ) flexure in the jejunum, 120 cm from the DJ flexure in the mesentery, and two at 140cm within the jejunum (Figure 2). The rest of the bowel length was unremarkable. All perforations were repaired in two layers and central venous access was maintained intraoperatively.

**Figure 3:**
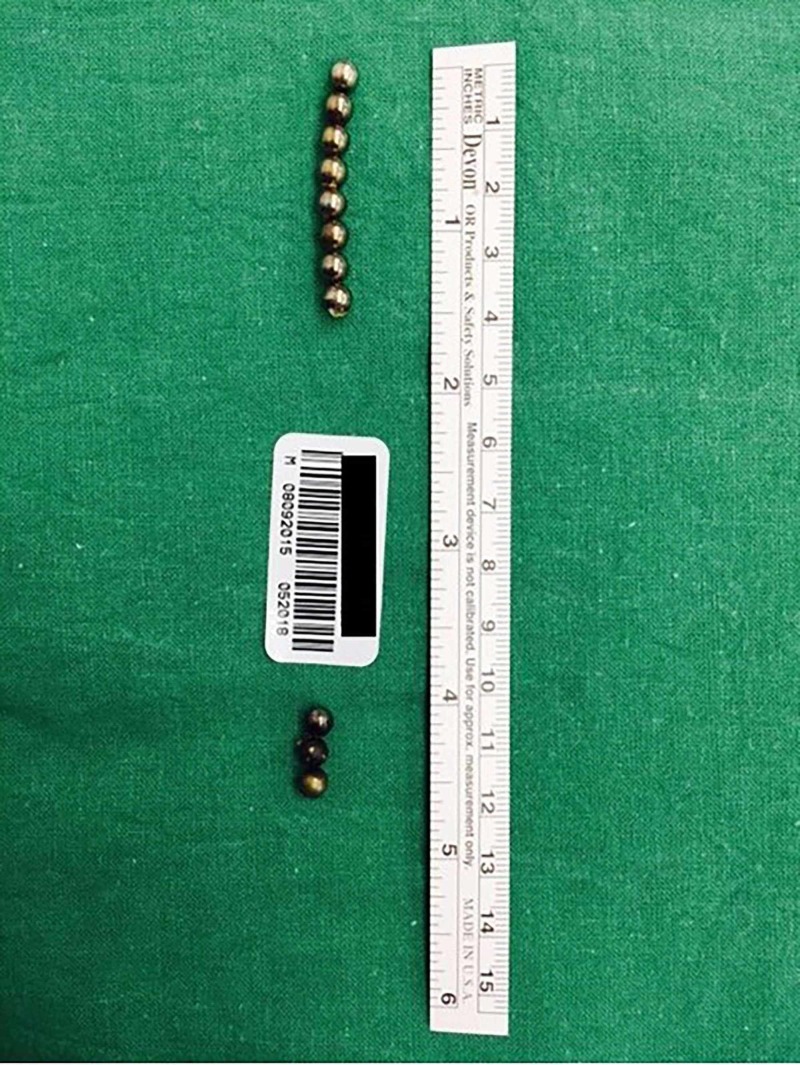
Three magnets retrieved from the stomach and eight magnets retrieved from in between small bowel loops

Altogether 11 spherical neodymium magnets were retrieved (Figure 3).

Minimal blood loss was encountered. Post-operatively, the child remained hemodynamically stable. He was kept nil per oral and started on total parental nutrition and tramadol infusion. He spiked fever on the third post-operative day, likely due to post-operative wound infection. Blood culture and swab cultures from the wound site were sent and antibiotics escalated accordingly. The child gradually improved and an oral diet was initiated which was well tolerated. Parenteral nutrition was discontinued, and pain management de-escalated. He was discharged on the ninth post-operative day, with all cultures revealing no significant growth of any microorganism.

**Figure 4:**
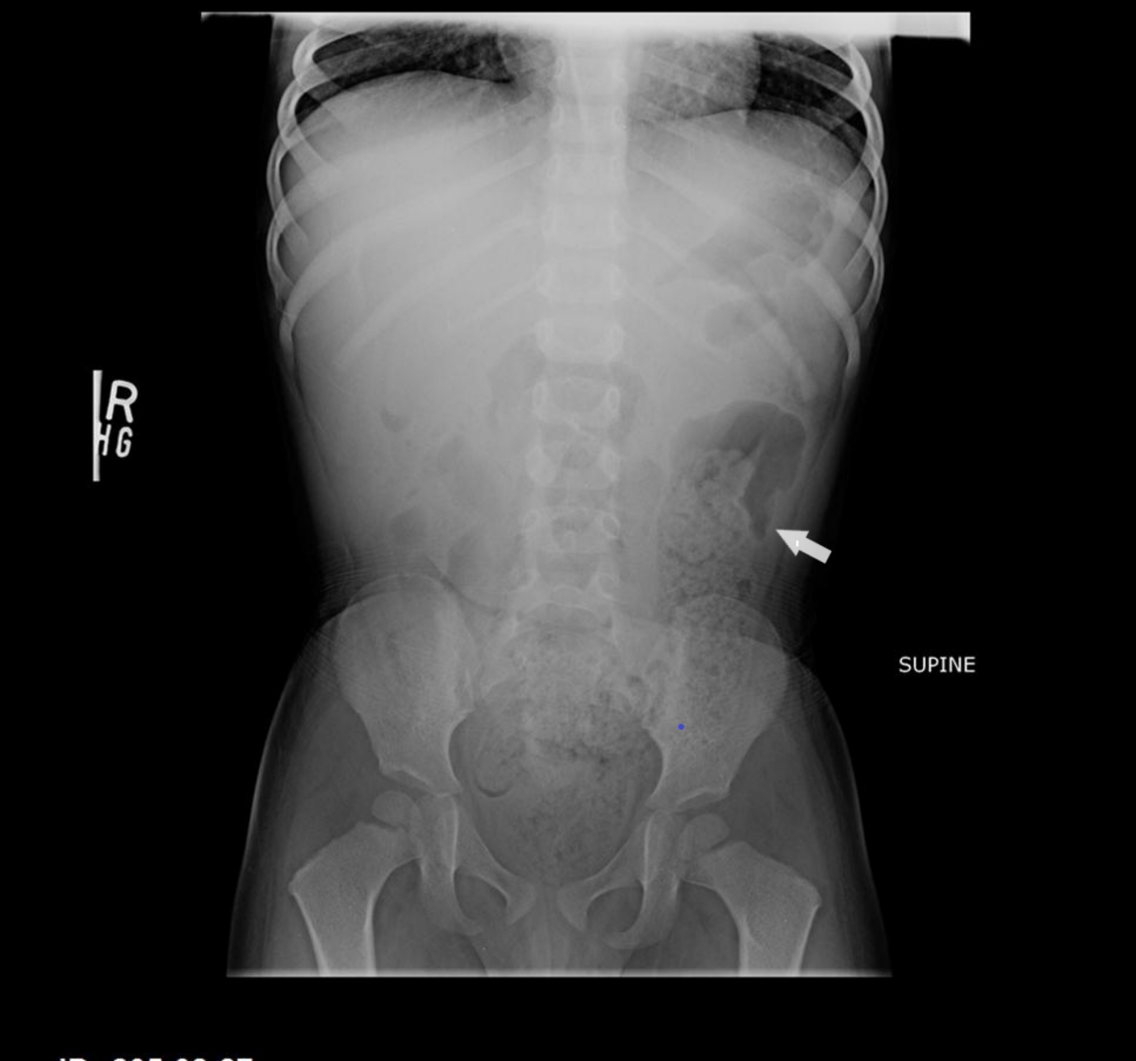
Repeat abdominal X-rays upon readmission Fecal loading seen in large bowel (arrow)

The child was later readmitted with persistent vomiting on the 11th post-operative day and managed as subacute intestinal obstruction. He was kept nil per oral, and an abdominal X-ray was repeated, which revealed fecal loading in large bowel (Figure 4). He then underwent therapeutic water-soluble contrast administration and subsequently improved. He was discharged on regular laxatives and he remained asymptomatic on subsequent follow-up visits.

## Discussion

Most cases of foreign body ingestion involve children between six months and three years of age. Fortunately, 80% of cases will usually have a spontaneous passage of the foreign body, with 10% to 20% requiring endoscopic removal and 1% requiring surgical intervention [3]. In 2011, the American Association of Poison Control documented 95,705 incidents of foreign-body ingestions by patients, younger than 20 years, with 74,725 occurring in children younger than at least five years [4]. In a recent 10-year retrospective review conducted in the USA, on an average, 16,386 patients present to the emergency department with possible magnet ingestion. Emergency department visits due to possible magnet ingestion have increased 8.5-fold from 2002 to 2011 with a 75% average annual increase per year in the USA. The majority of patients reported having ingested magnets were younger than five years [5]. This increase in incidence, as mentioned by Abbas et al., coincided with the growing availability of magnets, especially high-powered magnets (those containing neodymium) being sold as part of toys for children [5]. As a result of this trend, the Center for Disease Control and Prevention (CDCP) issued its first warning against these high-powered magnets in 2007.

Most foreign bodies, if small enough, are managed conservatively with watchful waiting and serial X-ray images. However, in cases of multiple magnet ingestion, X-ray imaging may be deceptive as magnets can appear as other less harmful inorganic foreign bodies (e.g., pearl) or may be indistinguishable from other metallic foreign bodies (e.g., coins, parts of jewelry) [1,6]. Thus unwitnessed cases of magnet ingestion can be a diagnostic dilemma, especially if the child is not symptomatic or presents with non-specific symptoms. Baily et al*.* reported a case of multiple magnet ingestion leading to gut perforation, after the performance of an MRI in a patient complaining solely of neck pain [7]. In reality, it is difficult to distinguish a magnetic foreign body from a metallic one in unwitnessed cases, as initial X-ray imaging can be misleading [8]. To decrease the risk further CDCP launched the Consumer Product Safety Act, which stipulated that any magnet manufactured or imported on or after April 1, 2015, must be large enough to decrease its ingestion hazard or the magnetic force must be lowered to a flux index of 50kG2 (37 times weaker than those commercially available in toys) [9].

**Figure 5:**
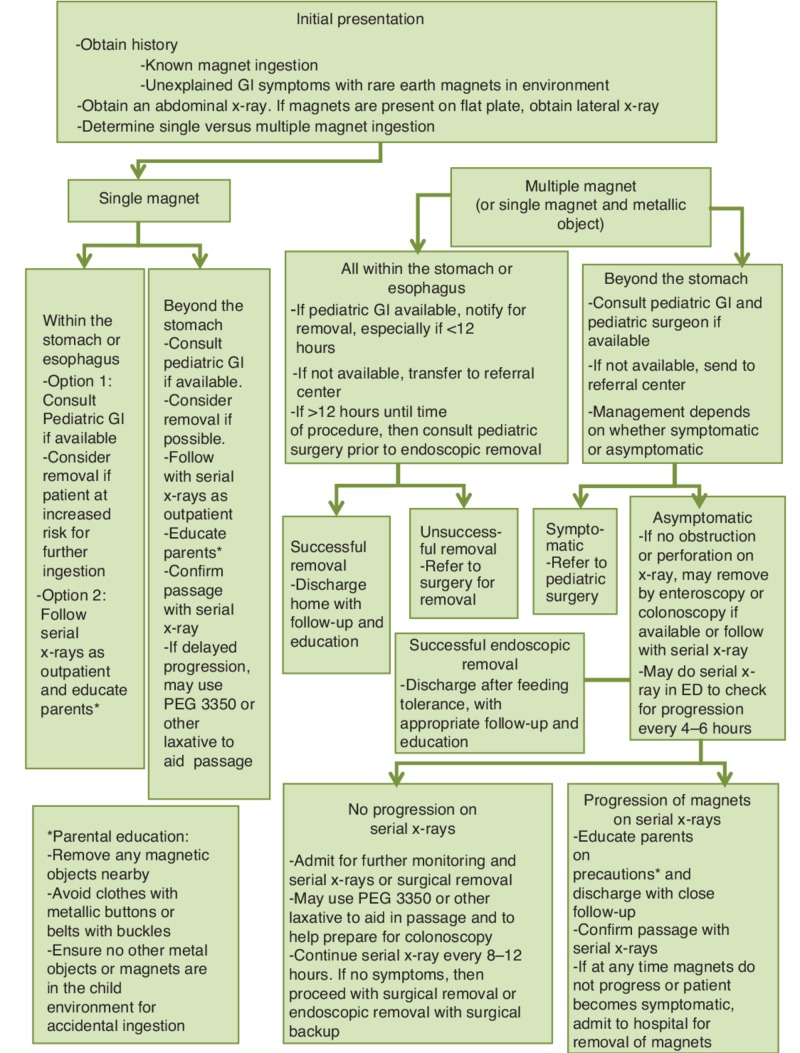
Proposed treatment protocol for pediatric magnet ingestion advised by NASPGHAN NASPGHAN, North American Societies of Pediatric Gastroenterology, Hepatology, and Nutrition

In our case, the ingestion was un-witnessed by parents, leading to delayed diagnosis and complications of gut perforation at multiple levels. Delayed diagnosis is a common concern. Another hindrance to effective management is that the initial diagnostic modality of X-ray imaging may mislead in exactly locating the magnetic foreign bodies. Essentially, all cases of presumed magnet ingestion, especially multiple, should undergo urgent surgical exploration to avoid associated morbidity such as pressure necrosis of the bowel, perforation, fistula formation, pneumoperitoneum with and without intra-abdominal or pelvic abscess formation, and in rare cases, intestinal volvulus [10]. The majority of foreign body obstructions tend to occur in children under three years of age, where a reliable history may be impossible to obtain. Thus, in children presenting with unexplained gastrointestinal symptoms, a low threshold for suspecting foreign body ingestion can be life-saving. In 2015 the North American Societies of Pediatric Gastroenterology, Hepatology, and Nutrition (NASPGHAN) devised a treatment algorithm for this purpose [11] (Figure 5). 

Waters et al*.* recently conducted a survey of surgical practices regarding magnet ingestions and recommended surgical management for multiple magnets located beyond the pylorus [12]. However, the NASPGHAN recommends non-operative management and advocate endoscopy if the patient is asymptomatic, with post-pyloric magnets. This difference in management practices could be explained by a third of the participants in the study by Waterset al. experiencing complications, such as fistulae and perforations [12]. If a single magnet versus multiple magnet ingestion cannot be definitively differentiated by history and radiographic findings in a stable patient, then inpatient treatment should be initiated, with the suspicion of ingestion of multiple magnets. 

Sola Jr. et al*. *conducted a multicenter, restrospective analysis of 89 cases of magnet ingestions in children. They compared children requiring abdominal surgery, with those managed conservatively. They found that there was no statistically significant difference between the two groups in terms of age, patient's gender or magnet location. However, patients were more likely to undergo surgery if they presented with abdominal pain (*p *< 0.01) and had multiple magnet ingestion. Similarly, patients were also more likely to require surgery if they had magnet ingestion accompanied by metallic foreign body ingestion (*p *< 0.01). In addition, the authors concluded that single magnet ingestion can be managed in the outpatient setting with a bowel regimen [13]. 

Magnetic forces of attraction inside visceral organs can be devastating. They allow the magnetic foreign bodies to attract one another, in between bowel loops. Once the two loops are in contact, pressure necrosis ensues, resulting in ischemic injury and subsequent perforation. Hussain et al. noted that ulceration and indentations of the mucosa may occur within eight hours of ingestion, which necessitates prompt removal [14]. Similarly, our case highlights the importance of prompt diagnosis and intervention to prevent complications. Greater physician awareness is also essential to suspect ingestion even in the presence of relatively non-specific symptoms.

## Conclusions

Magnet ingestion in a symptomatic patient has a high risk of leading to complications, especially if ingested with a metallic foreign body or if multiple magnets are ingested. A low threshold for surgical intervention in these patients prevents serious morbidity. Further prospective studies are needed for the development of a universal algorithm for the management of magnet ingestion.
